# The positive effect of perceived social support and moral resilience between moral injury and health-related productivity loss among emergency nurses

**DOI:** 10.3389/fpubh.2025.1678811

**Published:** 2025-11-05

**Authors:** Xiaoting Sun, Bingjie Wang, Meiyu Zhu, Daiying Wu, Miaomiao Yang, Chunmei Zhang

**Affiliations:** ^1^Graduate School, Tianjin University of Traditional Chinese Medicine, Tianjin, China; ^2^Emergency Department, Shandong Provincial Hospital Affiliated to Shandong First Medical University, Jinan, Shandong, China; ^3^State Key Laboratory of Experimental Hematology, National Clinical Research Center for Blood Diseases, Haihe Laboratory of Cell Ecosystem, Institute of Hematology & Blood Diseases Hospital, Chinese Academy of Medical Sciences and Peking Union Medical College, Tianjin, China; ^4^School of Nursing, Tianjin University of Traditional Chinese Medicine, Tianjin, China

**Keywords:** emergency nurses, moral injury, health-related productivity loss, perceived social support, moral resilience

## Abstract

**Background:**

Emergency nurses frequently experience moral injury (MI) arising from high-risk ethical conflicts, heavy workloads, and exposure to traumatic events, which can contribute to health-related productivity loss (HRPL). However, the underlying mechanisms remain unclear. Crucially, perceived social support and moral resilience may mediate this relationship by mitigating negative effects. Clarifying the mediating roles of perceived social support and moral resilience is essential to evaluate their influence on the relationship between MI and productivity loss, and to establish a model that explains this mechanism, thereby contributing to protecting nurses’ well-being and safeguarding patient care quality.

**Objective:**

This study aims to explore the relationship between MI and HRPL, and to examine the mediating roles of social support and moral resilience. These insights are of great significance for enhancing the physical and mental well-being of emergency nurses and improving the overall quality of medical care.

**Methods:**

A prospective cross-sectional survey was conducted among 483 emergency nurses from five tertiary hospitals across three provinces in mainland China between January and May 2025. The survey instruments included the General demographic questionnaire, Moral Injury Symptoms Scale-Health Professionals Version (MISS-HP), Rushton Moral Resilience Scale (RMRS), Perceived Social Support Scale (PSSS) and Stanford presenteeism scale-6 (SPS-6). Descriptive analysis and Pearson correlation analysis were performed using SPSS 29.0. The structural equation model was constructed with AMOS 29.0 software, and Bootstrap testing was conducted.

**Results:**

The results showed that moral injury directly affected Health-related productivity loss (*β* = 0.282, 95% CI [0.183, 0.382]). Perceived social support and moral resilience both played mediating roles in the relationship between MI and HRPL (*β* = 0.042, 95%CI [0.008, 0.076]) (*β* = 0.079, 95%CI [0.046, 0.117]). Perceived social support and moral resilience play chain mediating roles between MI and HRPL (*β* = 0.020, 95%CI [0.010, 0.034]). The structural model demonstrated good fit indices (CFI = 0.947, RMSEA = 0.045), indicating the robustness of the proposed model.

**Conclusion:**

Perceived social support and moral resilience jointly buffer the impact of MI on nurses’ health-related productivity. Interventions should therefore strengthen both support and resilience. For example, hospitals could implement peer-support groups or resilience workshops, and nurses could practice mindfulness or seek mentorship to bolster coping skills. These strategies may mitigate the negative effects of moral injury and improve productivity. However, as a cross-sectional survey relying on self-reported measures, this study may be subject to response bias, highlighting the need for future longitudinal research.

## 1 Introduction

In recent years, academic research has increasingly focused on moral injury (MI) among healthcare professionals, particularly emergency nurses, who are at heightened risk due to the inherent demands of their roles (1). MI arises not only from systemic pressures, such as chronic staffing shortages and limited medical resources, but also from situational stressors, including the urgency of life-or-death decisions and complex ethical dilemmas. The interplay between systemic and situational factors frequently gives rise to MI, which profoundly affects nurses’ physical and mental health as well as their professional performance (2). MI is an acute psychological and existential trauma marked by intense guilt, self-blame, and shame that occurs when individuals directly experience or witness events that violate their deeply held moral beliefs—referred to as Potentially Morally Injurious Events (PMIEs) (3). It arises from a profound conflict between a person’s moral code and real-life circumstances, whether through their own actions or by observing the actions of others (4). The contemporary framework posits a progressive pathway of PMIE → moral distress → MI. PMIEs initially precipitate moral distress. When such distress persists, remains unaddressed, and is coped with in a manner that contravenes an individual’s core values, it may culminate in MI (5, 6). Put differently, moral distress represents the psychological anguish experienced by an individual under moral duress (7). If it accumulates over an extended period and lacks effective intervention, it can serve as the precursor phase of MI. A more nuanced understanding distinguishes moral distress from MI. Moral distress refers to the psychological discomfort that arises following a PMIE, whereas MI represents a deeper and more enduring impairment that may develop when individuals respond to persistent moral distress in ways that are inconsistent with their value-based living (5). Therefore, while not all instances of moral distress lead to MI, chronic or severe distress serves as a significant pathway toward the development of MI. Emergency nurses are likely to be exposed to PMIEs due to the nature of their work (8). Emergency nursing is characterized by inherent ethical complexity, as nurses must frequently navigate morally challenging situations under urgent conditions. This often includes working in high-demand and high-stress environments for extended periods and caring for dying patients who might otherwise be saved if more resources were available (5, 6). It also involves making complex care-related decisions under extreme pressure, facing physical or verbal violence from patients or relatives, and engaging in emotionally charged conversations with grieving or angry family members (9, 10). These scenarios encompass both events that may directly constitute PMIEs and chronic workplace stressors, both of which can contribute to the development of MI (11). Evidence further confirms the widespread nature of MI. Rushton reported that nurses have the highest prevalence of MI among healthcare professionals, with a rate as high as 38.1% (12). A recent systematic review (2025) found that 38–65% of nurses experienced potentially morally injurious events, with emergency and critical care nurses having 1.72 times higher odds (95% CI: 1.38–2.14) of experiencing moderate-to-severe guilt and shame compared to general ward nurses (13). Persistent moral dilemmas have a significant impact on nurses’ physical and mental health, well-being, and professional commitment (14). For emergency nurses, prolonged exposure to moral distress often leads to physical symptoms such as insomnia, hypertension, and loss of appetite, and may even result in a lasting moral residue effect (15). Moral residue refers to the enduring emotional and ethical impact that accumulates over time when individuals repeatedly confront morally injury situations without fully addressing the associated pain (16).

If not identified and addressed timely, MI’s persistent psychological burden may not only exacerbate nurses’ emotional distress and mental health risks, such as depression, anxiety and PTSD (17), but also further transform into observable health-related presenteeism issue (1). HRPL, often termed hidden absenteeism or presenteeism, occurs when employees are physically present at work but unable to perform effectively due to physical or mental health problems. It results in reduced efficiency, impaired care quality, and increased risks to patient outcomes, particularly in emergency nursing, while also generating considerable economic costs (18). In addition, studies have shown that MI among emergency nurses is closely associated with burnout and compassion fatigue (1, 19, 20), and can undermine their professional identity and job satisfaction (4). These findings highlight the far-reaching negative impact of MI, which should not be overlooked.

A review of existing literature reveals that numerous studies have confirmed that the relationship between MI and impaired health productivity is not immutable (1, 13, 21). In this study, perceived social support and moral resilience were selected as mediating variables based on their established theoretical relevance and empirical support (22). Perceived social support is defined as an individual’s subjective perception and evaluation of the emotional and instrumental support available from leaders, family members, and friends (23). It is considered a key resource for mitigating the consequences of MI (20). Strong evidence suggests that high levels of perceived social support can effectively alleviate the consequences of MI. It enhances individuals’ sense of belonging and self-worth, promotes moral repair, and thereby mitigates the negative spillover effects of moral dilemmas on individuals’ health, productivity, and occupational functioning to a certain extent (24). Adequate social support serves as a protective buffer that maintains the well-being of emergency nurses, reinforces professional values, and sustains their capacity to provide safe and effective patient care (25). The latest systematic review (2025) indicates that adaptive coping strategies, such as social support, resilience training, and peer networks, have been shown to help improve the coping abilities of healthcare and emergency personnel and alleviate their distress (1). Previous research indicates that moral resilience serves as a crucial psychological resource enabling individuals to preserve their professional integrity in the face of moral adversity, including moral dilemmas, moral distress, or moral harm (26). Moral resilience refers to an individual’s ability to maintain or restore integrity and moral consistency when faced with ethical conflicts and psychological pressure. Existing conceptual analyses have suggested that the development of moral resilience depends on foundations such as ethics education, meaning construction, and value clarification, which equip individuals to navigate moral complexity more effectively, mitigate resulting moral distress, and maintain ethical standards under high-pressure conditions (26, 27). Empirical research further confirms that moral resilience, as a key internal psychological resource, serves as an important buffer against the psychological erosion caused by moral harm, helping to maintain the professional adaptability and stable work status of emergency nurses (28).

Therefore, perceived social support and moral resilience are considered important protective factors in mitigating the adverse effects of MI on health productivity (1, 12, 29)_._ Existing studies have examined mainly these variables in isolation. MI has been shown to increase presenteeism and reduce work performance (21, 30), and is negatively associated with both social support and moral resilience (24, 28). In contrast, social support and moral resilience can buffer stress, sustain productivity, and reinforce one another (22, 31, 32). However, few studies have investigated these variables within an integrated framework, particularly among emergency nurses. This is especially true for emergency nurses, who experience a high incidence of MI but for whom such research remains relatively scarce. To fill this gap, the present study employs the Job Demands–Resources (JD-R) Model and the Stress Buffering Hypothesis to systematically investigate how MI contributes to impaired health productivity, with particular attention to the mediating roles of perceived social support and moral resilience. This study aims to examine the dual protective effects of this pathway and to and to generate evidence that can guide the design of organizational and individual-level interventions to safeguard nurses’ well-being and care quality. The theoretical framework of this study draws on the following models.

### 1.1 Job demands–resources model

The JD-R model proposed by Bakker and Demerouti (33) emphasizes the dynamic balance between job demands and available resources. When job demands are excessive and personal or organizational resources are insufficient, employees are more likely to experience psychological exhaustion and burnout, leading to reduced work engagement and productivity loss. Conversely, adequate personal and external resources can buffer these negative effects, thereby maintaining mental health and performance. In emergency nursing, MI represents a high-intensity job demand that substantially depletes nurses’ psychological and emotional resources, increasing the risk of productivity loss. Perceived social support, as an external resource, provides emotional and informational assistance that alleviates isolation, whereas moral resilience, as an internal resource, enables nurses to recover from moral dilemmas and preserve professional integrity (34). Furthermore, a positive ethical climate and supportive leadership can strengthen these resources, mitigating the detrimental effects of MI and enhancing nurses’ capacity to deliver safe and effective patient care. This study holds significant theoretical value as it extends the JD-R model to the realm of MI. It elucidates both the mediating and chain-mediating mechanisms involving perceived social support and moral resilience. Furthermore, this research possesses practical implications that can inform multilevel interventions—such as supportive leadership, peer-support programs, resilience training, and individual coping strategies—that aim to mitigate productivity loss, safeguard the health and well-being of emergency nurses, and ultimately enhance the quality of patient care.

### 1.2 Buffering hypothesis

**Figure 1:**
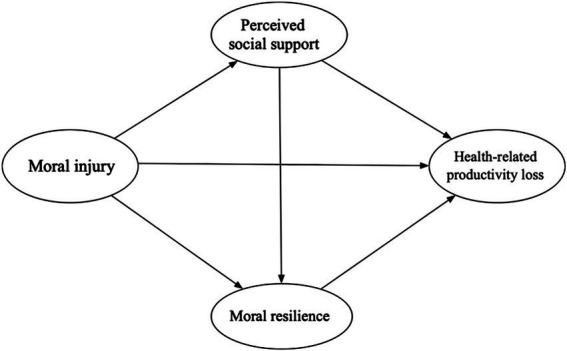
The theoretical model of this study.

The buffering hypothesis posits that social support can reduce individuals’ perceived stress, thereby helping to maintain physical and mental health (35). Internal resources such as resilience, optimism, and self-efficacy have likewise been shown to mitigate the adverse effects of stress and facilitate recovery from illness or major life events (35). In professions with high moral demands, moral resilience is considered a crucial internal resource that enables individuals to cope with ethical dilemmas and psychological conflicts, allowing them to preserve value consistency and psychological balance when facing challenges. Rushton’s research further demonstrated that moral resilience can significantly buffer the negative effects of moral distress on mental health and reduce the risk of burnout under high moral pressure (28). Building on this foundation, the present study integrates the JD-R model with the buffering hypothesis to examine how perceived social support and moral resilience, as key external and internal resources, moderate the impact of MI on the health and productivity of emergency nurses (Figure 1).

### 1.3 Research hypotheses

Based on the above theory, the following hypotheses are proposed:

*Hypothesis 1*: MI will significantly predict HRPL.

*Hypothesis 2*: Perceived social support mediates the relationship between MI and HRPL.

*Hypothesis 3*: Moral resilience mediates the relationship between MI and HRPL.

*Hypothesis 4*: Perceived social support and Moral resilience act as chain mediators between MI and HRPL.

## 2 Methods

### 2.1 Study design

This study employed a cross-sectional, online questionnaire survey design. The results were reported according to the Strengthening the Reporting of Observational Studies in Epidemiology (STROBE) guidelines (36) (Supplementary File 1).

### 2.2 Participants, setting, sampling

In this study, emergency department nurses from five tertiary hospitals in Beijing, Tianjin, and Jinan, China, were selected from January to May 2025 using convenience sampling. The inclusion criteria for participants were: (a) no current or past diagnosis of mental illness or drug or alcohol dependence, (b) holding a valid nurse practice license and having experience in the emergency department, and (c) voluntary participation in the study. Participation is voluntary and no rewards are offered to participants. Exclusion criteria were: (a) nurses who are not engaged in clinical work (e.g., nurses in administrative positions), (b) nurses who are absent due to maternity leave, sick leave, study leave or other similar reasons, and (c) nurses currently undergoing standardized training or rotating through departments, trainee nurses, probationary nurses, retired or rehired retired nurses.

In accordance with Kendall’s criteria, the sample size should be 5–10 times larger than the number of items (37). This study uses 4 scales with a total of 44 items, considering a loss of 0.20 samples. The minimum sample size was 264, and the larger the sample size, the more stable the mediating effect. A total of 501 participants were collected. During this time, participants can contact researchers via WeChat if they have any questions. Once the survey is complete, the collected data will be collated. After data cleaning, 19 participants were excluded from questionnaires with a response time of less than 100 s or all the same or regular options in the questionnaire, resulting in 19 participants being excluded, and the final 482 completed questionnaires (response rate: 96.21%) were available for analysis.

### 2.3 Instruments

#### 2.3.1 General demographic questionnaire

Demographic data of participants included gender, age, marital status, professional title, working years, educational level, labor and personnel relations, average monthly income (RMB), average number of night shifts, and participation in hospital ethics course.

#### 2.3.2 Moral injury symptoms scale-health professionals version

The Moral Injury Symptoms Scale-Health Professionals Version (MISS-HP) for Medical Personnel was originally developed by Mantri et al. (38). This study used the Chinese version of the MISS-HP, which was translated and adapted by Zhizhong et al. (39), which includes 10 items divided into 3 dimensions: shame and guilty, mistrust, and forgiveness. A visual analog scale with response options ranging from 1 (strongly disagree) to 10 (strongly agree) was used for each of the scale’s 10 items. To lessen response bias, six of the items are written negatively and four positively. A score between 10 and 100 is calculated once the positively phrased items (5, 6, 7, and 10) have been recoded. Scores of 50 or higher on the scale have been shown to indicate significant difficulties with social and occupational functioning in this population. Higher values indicate greater MI. The Chinese version of MISS-HP as proven to be reliable and effective (*α* = 0.930). In this study, the alpha score was 0.950, indicating satisfactory internal reliability. Generally, a Cronbach’s alpha coefficient >0.7 is considered acceptable (40).

#### 2.3.3 Perceived social support scale

Perceived Social Support Scale (PSSS) was originally developed by Zimet et al. (41). This study adopts the Chinese translation and revision of PSSS by Jiang et al. (1999) (42). It is used to assess the level of social support perceived by individuals. This scale consists of 3 dimensions and 12 items: 4 items for family support, 4 items for friends support, 4 items for other support. This scale is based on a 7-point Likert scale, 1 point stand for very disagree and 7 points stand for very agree. The total score ranges from 12 to 84, with higher scores indicating higher levels of perceived social support. The Cronbach’s alpha coefficient of this scale is 0.900. The Chinese version of PSSS has proven to be reliable and effective (*α* = 0.961). In this study, the alpha score was 0.975, indicating satisfactory internal reliability.

#### 2.3.4 Rushton moral resilience scale

Rushton Moral Resilience Scale (RMRS) was originally developed by Heinze et al. (43). This study adopts the Chinese translation and revision of RMRS by Qingqing et al. (44). The Chinese version of the scale consists of 16 items in 4 dimensions of responses to moral adversity (4 items), moral efficacy (4 items), relational integrity (5 items), and personal integrity (3 items). It uses a 4-point Likert scale, ranging from 1 (“Disagree”) to 4 (“Agree”). Among them, items 2, 4, 5, 6, 8, 10, 11, 13, 14, 15, and 16 are reverse-scored. This means that when scoring in reverse, 1 point is converted to 4 points, 2 points to 3 points, 3 points to 2 points, and 4 points to 1 point. The total score is calculated by summing the responses to all 16 items after reverse-scoring the appropriate items, with possible scores ranging from 16 to 64. Higher scores indicate greater MR among nurses. The Chinese version of RMRS has proven to be reliable and effective (*α* = 0.840). In this study, the alpha score was 0.968, indicating satisfactory internal reliability.

#### 2.3.5 Stanford presenteeism scale-6

Stanford presenteeism scale-6 (SPS-6) originally developed by Koopman et al. (45). This study adopts the Chinese translation and revision of SPS-6 by Zhao et al. (46). SPS-6 has become a widely adopted tool to assess the impact of health status on individual productivity. The scale consisted of 6 items in 2 dimensions. Each item was rated on a 5-point Likert scale, ranging from 1 to 5 from complete disagreement to complete agreement, respectively, with entries 5 and 6 reversed, for a total score of 6 to 30. Items 1 to 4 are forward scoring, and the last 2 items are reverse scoring. Higher scores indicated a greater loss of health-related productivity due to sickness presenteeism. The scale of Cronbach’s *α* was 0.860. The Chinese version of SPS-6 exhibited sufficient reliability and validity. The results showed that the Cronbach’s α coefficients were 0.936, in this study, the alpha score was 0.903.

### 2.4 Ethical considerations

The study was approved by the Ethics Committee of the Affiliated Hospital of Shandong Provincial Hospitals (SWYX: NO.2024–735). Data collection is carried out on the principles of anonymous and informed consent. The initial page of the online survey presented participants with standardized information regarding the study’s objectives, their rights as participants, and the procedures for withdrawal. Participants were informed that they had the right to withdraw from the study at any time without providing a reason. By choosing to continue their participation in the survey, participants implicitly consented to take part in the research.

### 2.5 Data collection

Before the formal investigation, a pilot test was conducted with 20 nurses. The purpose of this pre-experimental phase was to identify any ambiguities in the questionnaire’s questions, address and resolve these issues, and determine the time required to complete the survey. The pilot test confirmed the questionnaire’s clarity and completeness based on nurses’ feedback, and showed that it could be completed within 5–10 min, demonstrating its feasibility for formal use. The participants from the pilot test were not included in the formal study. Data collection began after the study was authorized by the Ethical Review Committee. The questionnaires were distributed via WenJuanXing platform^1^, a widely used online survey platform in China. After obtaining consent, the head nurse will distribute a questionnaire link via the WeChat platform to emergency department nurses who meet the inclusion criteria, in order to conduct the survey. The homepage of the questionnaire included a standardized explanation. All participants provided informed consent and were informed that they could withdraw at any time. To ensure data integrity, all questions were mandatory to avoid missing data. Additionally, only one questionnaire could be submitted from the same IP address to prevent duplicate entries. All questionnaires were completed anonymously. After the questionnaires were collected, they were manually reviewed. Questionnaires with response times less than 100 s or those with identical or patterned answers for all questions were excluded.

### 2.6 Data analysis

The questionnaire results were directly exported from the backend of WenJuanXing platform. All data analyses were performed using SPSS 29.0 and AMOS 29.0. The data met the normality assumption and were tested for normality using the Shapiro–Wilk test, with a *p*-value greater than 0.05, indicating that the data followed a normal distribution. Graphics were used to construct a mediation model with MI as the independent variable. Frequency and percentage were used to describe the general characteristics of the participants. The relationship between the four variables was examined using Pearson correlation analysis Cronbach’s alpha coefficient was used to assess the internal consistency of the scales. The validation factor analysis of the samples in this study indicated an acceptable model fit. Mediation analysis was used to explore the mediating role of perceived social support and moral reliance in the relationship between emergency nurses’ MI and HRPL. In addition, AMOS 29.0 was applied to develop the model and analyze variable associations and parameters. This study employed chi-square tests to assess model fit, utilizing *χ*^2^/df, the Comparative Fit Index (CFI), Adjusted Goodness-of-Fit Index (AGFI), Root Mean Square Error of Approximation (RMSEA), Goodness-of-Fit Index (GFI), Incremental Fit Index (IFI), and Tucker–Lewis Index (TLI) to evaluate the overall adequacy of the hypothesized model. The smaller the *χ*^2^/df value, the better the model fit. A smaller RMSEA indicates a better-fitting model. For GFI, TLI, CFI, and AGFI, values range from 0 to 1, with values closer to 1 indicating a better fit (47). Finally, Bootstrap method was used to calculate the 95% CI by repeated sampling 5,000 times, if none of the results contained 0, the mediation effect was significant. Differences were considered statistically significant at *p* < 0.05 (48). The indirect effect was calculated as the total effect minus the direct effect, and the indirect effect was equal to the product of the standardized path coefficients of the mediating variables (Table 1).

**Table 1:** Sociodemographic characteristics of the participants (*n* = 482).

Characteristics	Categories	Number percentage (%)
Gender	Male	97(20.1)
	Female	385(79.9)
Age (years)	≤30 years	217(45.1)
	31–40 years	175(36.1)
	41–50 years	79(16.4)
	> 50 years	12(2.4)
Marital status	Married	306(63.5)
	Unmarried	158(32.8)
	Divorced/Widowed	18(3.7)
Professional title	Nurse	120(24.9)
	Senior nurse	154(32.0)
	Supervisor nurse	143(29.7)
	Deputy/Director nurse	65(13.4)
Working years	< 3 years	144(29.9)
	3–5 years	102(21.1)
	> 5 years	236(49.0)
Educational level	Below bachelor’s degree	55(11.5)
	Bachelor’s degree	335(69.5)
	Above bachelor’s degree	92(19.0)
Labor and personnel relations	Contract system	200(41.5)
	Personnel agency	103(21.4)
	Formally in the compilation	179(37.1)
Average monthly income (RMB)	<5,000	60(12.4)
	5,001–7,000	90(18.7)
	7,001–9,000	98(20.3)
	9,001–10,000	89(18.5)
	>10,000	145(30.1)
Average number of night shifts (pieces)	0	100(20.7)
	1–3	85(17.6)
	4–6	108(22.4)
	7–9	116(24.1)
	≥10	63(13.1)
Participation in hospital ethics course	No	138(28.6)
	Yes	344(72.4)

## 3 Results

### 3.1 Sociodemographic characteristics of the participants (*n* = 482)

Table 2 presents the demographic characteristics of the participants. A total of 482 nurses were included in this study. Most participants were female nurses (385, 79.9%) age: 33.88 ± 7.98 years, were married (306, 63.5%), were senior nurses (154, 32.0%), and a significant proportion of emergency nurses have been working for more than 5 years (236, 49.0%). It shows that nearly half of the research subjects have extensive work experience. The majority held Bachelor’s degrees (335, 69.5%) and had received training in moral and ethical issues in the hospital (344, 72.4%). In terms of employment methods, (200, 41.5%) are contract-based, (103, 37.1%) are permanent staff, and (179, 21.4%) are other forms, demonstrating the diversity of employment methods.

**Table 2:** Descriptive statistics and correlation analysis.

Variable	*M*	SD	MISS-HP	PSSS	RMRS	SPS
MISS-HP	50.04	19.645	1			
PSSS	38.17	13.839	−0.399**	1		
RMRS	53.71	16.408	−0.337**	0.338**	1	
SPS-6	16.08	5.436	0.389**	−0.375**	−0.282**	1

M, mean; SD, standard deviation; **correlation is significant at the 0.01 level (2-tailed).

### 3.2 Correlations between MI, perceived social support, moral resilience, and HRPL

Table 2 shows the correlation matrix and Pearson’s correlation analysis of MI, perceived social support, moral resilience, and HRPL. It can be noted that the mean scores of MI, perceived social support, moral resilience, and HRPL were 50.04 (19.645), 38.17(13.839) and 16.08 (5.436) respectively. MI was significantly negatively correlated with moral resilience moral resilience (*r* = −0.399, *p* < 0.01), significantly negatively correlated with perceived social support, (*r* = −0.337, *p* < 0.01), and significantly positively correlated with HRPL (*r* = 0.389, *p* < 0.01), indicating that higher MI levels are associated with lower perceived social support and moral resilience levels, while HRPL levels are higher. Perceived social support showed a significant positive correlation with moral resilience (*r* = 0.338, *p* < 0.01) and a significant negative correlation with HRPL (*r* = −0.375, *p* < 0.01), indicating that perceived social support, and moral resilience exhibit a synergistic trend of change, while perceived social support perceived social support, and HRPL show an opposite trend of change. The significant negative correlation between moral resilience and HRPL (*r* = −0.282, *p* < 0.01) further reveals the inverse association between these two variables. Overall, the direction of correlations among the variables aligns with the research hypotheses and all reach a highly significant level, providing preliminary data support for the construction of subsequent structural equation models.

### 3.3 Mediating effects of MI, perceived social support, moral resilience, and HRPL

**Figure 2:**
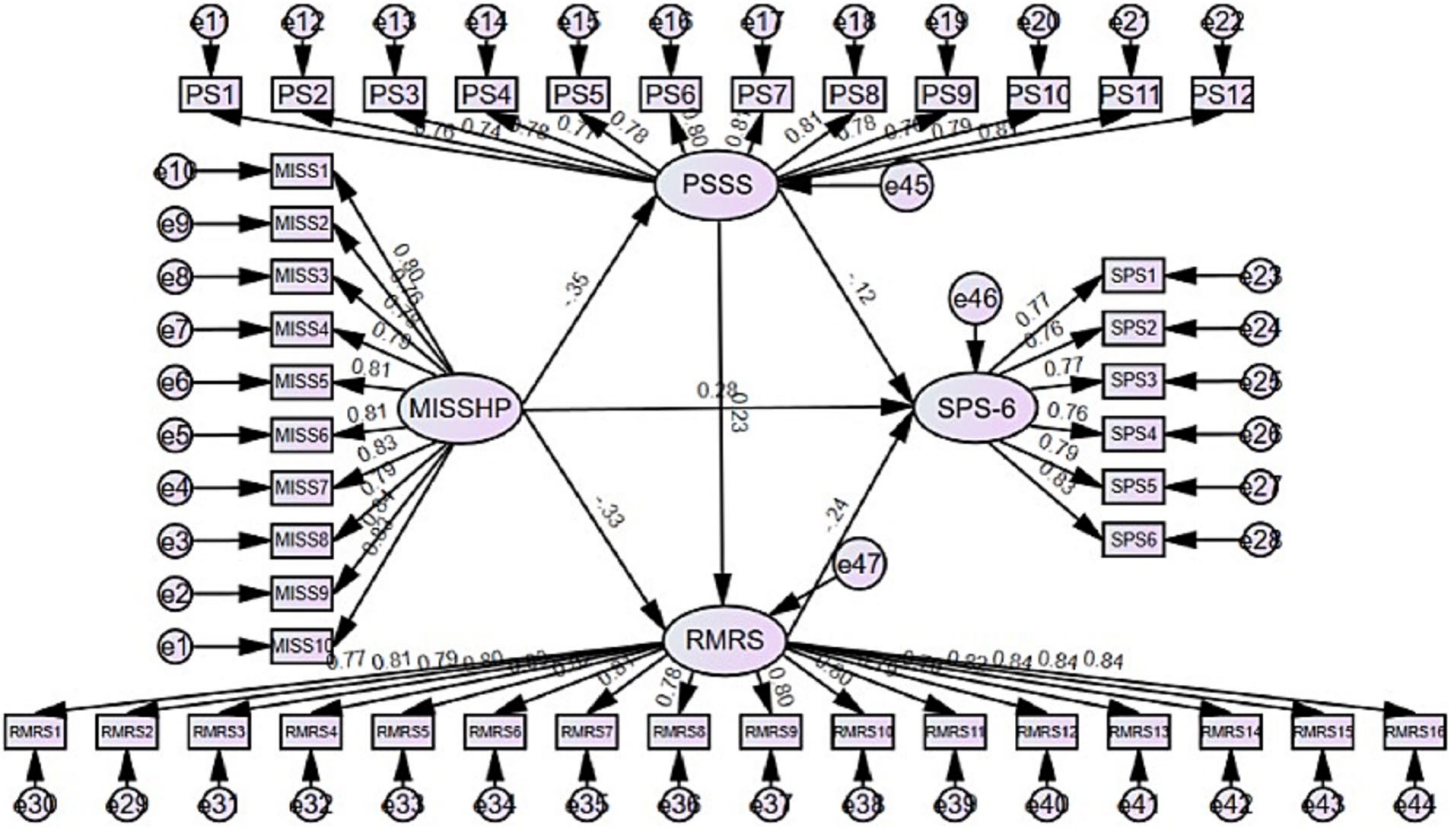
The mediating roles of MI, perceived social support, moral resilience, and HRPL.

This study used AMOS 29.0 software to test for the mediating effect (49). The model fitting index of the corrected model is acceptable: *χ*^2^/df (Chi-square to degrees of freedom ratio) = 1.995, RMSEA (Root Mean Square Error of Approximation) = 0.045, SRMR (Standardized Root Mean Square Residual) = 0.032, AGFI (Adjusted Goodness of Fit Index) = 0.852, GFI (Goodness of Fit Index) = 0.866, IFI (Incremental Fit Index) = 0.947, CFI (Comparative Fit Index) = 0.947, and TLI (Tucker–Lewis Index) = 0.944. Table 3 summarizes the final model fit indices; all values met or exceeded recommended thresholds, indicating an acceptable fit. The mediation model constructed is shown in Figure 2.

**Table 3:** Model-fitting standard and fitting index of the final model.

Model fit	Model-fitting index	Model-fitting standard
CMIN/DF	1.995	1–3
RMSEA	0.045	<0.08
SRMR	0.032	<0.05
NFI	0.899	>0.8
TLI	0.944	>0.8
GFI	0.866	>0.8
AGFI	0.852	>0.8
IFI	0.947	>0.8
CFI	0.947	>0.8

Table 4 shows the results of the path analysis of the causal relationships between variables. All path coefficients passed the significance test (*p* < 0.05 or *p* < 0.001), and the constructed structural model has good explanatory power. Specifically, MI had a significant negative impact on perceived social support (*β* = −0.241, *p* < 0.001) and also had a significant negative impact on moral resilience (*β* = −0.155, *p* < 0.001), indicating that an increase in moral injury levels simultaneously inhibits the development of both perceived social support and moral resilience. Perceived social support has a significant positive effect on moral resilience (*β* = 0.160, *p* < 0.001), indicating that higher levels of perceived social support are associated with greater moral resilience. This finding is consistent with the positive correlation observed between the two variables. In the influence path on the negative effect of perceived social support (*β* = −0.074, *p* < 0.05) and the positive effect of MI (*β* = 0.121, *p* < 0.001) coexist. Notably, Moral resilience has a significant negative impact on HRPL (*β* = −0.218, *p* < 0.001), with the absolute value of its path coefficient exceeding those of other direct influence paths, suggesting that moral resilience may play a crucial mediating role in the variable relationships. The 95% confidence intervals for all paths do not include 0, further validating the significance of these path effects.

**Table 4:** Path analysis.

Path	Estimate	S. E.	C. R.	*P*
MISS-HP → PSSS	−0.241	0.033	−7.300	<0.001
MISS-HP → RMRS	−0.155	0.022	−6.942	<0.001
PSSS → RMRS	0.160	0.032	4.970	<0.001
PSSS → SPS-6	−0.074	0.031	−2.419	0.016
MISS-HP → SPS-6	0.121	0.022	5.445	<0.001
RMRS → SPS-6	−0.218	0.047	−4.667	<0.001

MISS-HP, moral injury; PSSS, perceived social support; RMRS, moral resilience; SPS-6, health-related productivity loss.

Table 5 presents the effect decomposition of the mediation model of MI on HRPL based on the bootstrap sampling method, showing the specific parameters of the total effect, direct effect, and three indirect effect paths. The total effect of MI on HRPL is significant (*β* = 0.422, 95% CI = 0.332–0.500). The direct effect of MI on HRPL was dominant (*β* = 0.282, 95% CI = 0.183–0.382), accounting for 66.8% of the total effect.

**Table 5:** Bootstrap analysis of the mediating model.

Effect	Path	*β*	The size of effect	BootSE	95%CI Lower	95%CI Upper	*P*
Total	MISS-HP → SPS	0.422	—	0.043	0.332	0.5	0.001
Direct	MISS-HP → SPS	0.282	66.80%	0.051	0.183	0.382	0.001
Indirect1	MISS-HP → PS → SPS	0.042	10.00%	0.018	0.008	0.076	0.013
Indirect2	MISS-HP → RMRS → SPS	0.079	18.70%	0.018	0.046	0.117	0.001
Indirect3	MISS-HP → PS → RMRS → SPS	0.020	4.70%	0.006	0.010	0.034	0.001
	Total Indirect	0.140	33.20%	0.025	0.095	0.191	0.001

In the indirect effect path, indirect effect 1: perceived social support has a mediating effect between MI and HRPL, that is, ‘MI → perceived social support → HRPL’. The mediating effect value is 0.042, accounting for 10.0% of the indirect effect; indirect effect 2: perceived social support has a mediating effect between MI and HRPL, that is, ‘MI → moral resilience → HRPL’. The mediating effect value is 0.079, accounting for 18.7% of the indirect effect. The mediating effect of moral resilience was more significant, with an effect size approximately 1.88 times that of the perceived social support mediating effect, serving as an important indirect channel through which MI influences; Indirect effect 3: perceived social support and moral resilience play chain mediating roles between MI and HRPL, with the pathway being ‘MI → perceived social support→ moral resilience → HRPL’ and the chain mediating effect value is 0.020, accounting for 4.7% of the total indirect effect.

The total indirect effect *β* value was 0.14, accounting for 33.2% of the total effect, further validating the multiple mediating effects of perceived social support and moral resilience in the relationship between MI and HRPL. Among these, moral resilience alone contributed nearly one-fifth of the total effect, highlighting its key role in the mediating mechanism.

## 4 Discussion

This study identified four key findings. First, MI significantly predicts HRPL and negatively impacts perceived social support and moral resilience. Second, perceived social support mediates the relationship between MI and HRPL. Third, moral resilience mediates the relationship between MI and HRPL. Finally, perceived social support and moral resilience sequentially mediate the relationship between MI and HRPL. This chain-mediation mechanism was validated through structural equation modeling, contributing new insights to the literature. By integrating the JD-R model and the buffering hypothesis, we demonstrated how external (perceived social support) and internal (moral resilience) resources dynamically interact to buffer the effects of MI. This study offers a novel contribution by shifting the focus from psychological outcomes of MI to HRPL, thereby providing a new finding on its impact. By empirically confirming the dual and chain-mediating roles of perceived social support and moral resilience, the findings clarify the mechanisms through which protective resources operate, extend the JD-R model to the MI context, and provide actionable evidence for multilevel interventions that enhance support and resilience to safeguard both nurses’ well-being and patient care quality.

### 4.1 Mediating roles of perceived social support and moral resilience

This study found that MI was strongly negatively correlated with both perceived social support and moral resilience among emergency nurses, yet it was positively correlated with impaired health productivity. These results confirm Hypothesis 1. This harm not only directly threatens individual mental health (e.g., increased risks of depression, anxiety, and post-traumatic stress disorder) but also significantly impairs their ‘health productivity’ (presenteeism), i.e., the decline in work efficiency, quality, and overall functionality resulting from working while ill or in poor health (21). It also imposes substantial economic losses on healthcare organizations, with costs far exceeding the visible direct costs (30). This is consistent with previous research findings (50, 51). Evidence from studies conducted during the pandemic further highlights the severity of this issue: Kinman et al. (52) noted in a large-scale, multi-center study involving emergency nurses in the UK that the level of MI among nurses during the COVID-19 pandemic was positively correlated with presenteeism. For every 10-point increase in MI scores, the incidence of presenteeism-related errors (such as medication errors and record omissions) increased by 18% (52). It is evident that working while ill significantly increases the incidence of nursing errors such as medication errors and record omissions. Therefore, the validation of Hypothesis 1 is not only statistically significant but also has practical clinical and practical management significance, suggesting that MI is an ‘invisible killer’ of emergency nurses’ occupational health and patient safety, requiring early identification and systematic intervention.

The results of this study indicate that perceived social support and moral resilience significantly mediate the relationship between MI and impaired health productivity, explaining 10.0 and 18.7% of the total effect, respectively. Notably, these mediating paths are empirically supported by significant paired correlations, while perceived social support and moral resilience are negatively correlated with impaired health productivity, supporting Hypotheses 2 and 3. This aligns with previous research findings (50, 53, 54). Furthermore, it is not surprising to find a positive correlation between social support and moral resilience, as these concepts are known protective factors that improve nurses’ moral behavior and health productivity (22, 25, 55). For example, findings from the Chinese Nurse Health Cohort Study further confirm the importance of higher levels of perceived social support in buffering the negative effects of work-related stressors. When nurses perceive positive social support, manifested as support from colleagues, leaders, or family members, it can significantly mitigate the adverse effects of MI on health and productivity losses (42). First, high levels of social support help individuals proactively cope with work stress and challenges and effectively utilize internal and external resources (56). In particular, when emergency nurses are confident in their abilities, they tend to be more satisfied with their jobs, have good mental health, are more proactive in seeking help and support from others at work, and are more likely to perceive the presence of social support (57). The process of increasing emergency nurses’ confidence and sense of responsibility at work is also a process of gaining support from the social and work environment. In this process, nurses can more proactively address work challenges and reduce the impact on their health and work efficiency (58). These findings are consistent with previous evidence suggesting that social support can buffer the negative effects of occupational stress on well-being and performance (22, 59) and are empirically supported by our study, highlighting the importance of social support in reducing the adverse consequences of MI on nurses’ work outcomes (53). Secondly, a high level of social support helps improve individuals’ mental health, enhances emergency nurses’ sense of belonging and loyalty, thereby encouraging them to actively engage in their work and reducing the extent of damage to health productivity (60). Therefore, this evidence further reinforces the feasibility of improving nurses’ understanding of social support as a viable intervention to mitigate the consequences of MI.

At the same time, moral resilience reduces the impact of damage on healthy productivity (61). This result supports the findings of Gülhan Erkuş that leadership and colleague support are factors influencing moral resilience, and that higher moral resilience is associated with greater leadership and colleague support (62). The reason for this may be that moral resilience, as a positive psychological resource, helps individuals maintain internal value consistency when faced with moral dilemmas, enhances their ability to cope with negative emotions such as guilt and shame, and thereby reduces psychological exhaustion and behavioral withdrawal caused by moral conflicts. Additionally, nurses with higher moral resilience are typically more adept at seeking external support resources (peer discussions or ethical consultations), enabling them to achieve emotional repair and value clarification in stressful situations. Therefore, higher moral resilience not only directly enhances nurses’ psychological resilience in addressing moral dilemmas but also indirectly mitigates the negative impact of moral harm on mental and physical health and productivity by leveraging other support resources.

### 4.2 Chain-mediating effect of perceived social support and moral resilience

The most important finding is the chain mediating effect of perceived social support and moral resilience on the relationship between MI and HRPL, hypothesis 4 was supported. Study variables are dynamically interrelated and mutually influential rather than isolated constructs (63). This indicates that MI not only directly causes damage to health productivity, but also indirectly exacerbates damage to health productivity by consuming nurses’ ability to provide supportive understanding, social support, and moral resilience (34, 61). Emergency room nurses frequently encounter situations such as resource shortages and ethical conflicts in high-pressure environments, making it difficult for them to fully uphold their core moral beliefs (64). This can lead to profound guilt, shame, and psychological conflict, resulting in severe MI (65, 66). When this MI accumulates to a certain extent, it not only directly erodes nurses’ mental health and work performance but also reduces their perception of support from colleagues and the organization, weakens the role of external social support, and continuously depletes their internal moral resilience, making it harder for them to self-heal from emotional trauma (67). Ultimately, this results in greater negative impacts on health and productivity (68). This highlights the need for clinical managers to focus on ethical support and psychological adjustment for emergency nurses, fostering a supportive environment and resilience-building to mitigate the adverse consequences of MI. From a theoretical perspective, this finding supports the ‘health depletion pathway’ in the Job Demands-Resources JD-R model: MI acts as an extreme psychological demand, rapidly depleting nurses’ internal resources (social support and moral resilience), leading to energy depletion and impaired functioning. Emergency nurses, operating in resource-constrained environments where life-and-death decisions are frequent, are highly prone to intense guilt and shame due to the inability to provide optimal care. This persistent psychological conflict not only directly undermines their focus and decision-making efficiency but also amplifies the risk of presenteeism through a ‘resource depletion-cognitive rumination’ spiral.

However, perceived social support is not merely a passive target of consumption; it is also a critical external resource that can buffer the effects of MI as part of a larger project to study factors influencing nurses’ work performance and mental health. Emergency nurses with high levels of perceived social support are more likely to exhibit greater moral resilience (69). When social support and moral resilience are included as mediating variables, moral harm and impaired health productivity are reduced. These findings may be attributed to the fact that perceived social support provides external emotional and resource support, helping emergency nurses to cope more effectively with moral conflicts and distress. Meanwhile, the stress buffering hypothesis suggests that moral resilience, as an internal resource, enhances emergency nurses’ ability to manage guilt and shame while maintaining moral integrity when faced with ethical dilemmas. In summary, higher levels of social support may enhance moral resilience, thereby buffering the negative psychological impacts of MI and ultimately reducing its adverse effects on nurses’ health and productivity. This result can be explained by the complementary roles of external and internal resources in buffering the negative impacts of MI. Furthermore, recent research emphasizes that perceived social support enhances nurses’ ability to maintain integrity in moral dilemmas by fostering moral resilience (70). In turn, moral resilience is an internal psychological resource that enables nurses to manage guilt, shame, and moral dissonance more effectively. Therefore, nursing managers must not only maintain positive nurse–patient relationships but also build harmonious relationships with hospital colleagues. Effective communication among medical staff, nurses, and patients can enhance emergency nurses’ positive perception of hospital support.

### 4.3 Strengths of this study

First, by investigating MI and HRPL among emergency nurses, this study makes an important contribution to occupational health and nursing management. Second, the use of validated measurement instruments ensures the reliability and validity of the core variables, thereby strengthening both external validity and persuasiveness. Third, as one of the few empirical studies to systematically examine the mediating roles of perceived social support and moral resilience, this research goes beyond descriptive approaches by exploring the moderating and mediating functions of internal factors within this relational chain. Finally, through integrating the JD-R model and the buffering hypothesis, the study proposes that moral resilience and perceived social support, as critical internal and external resources, may explain the mechanisms linking MI to impaired health productivity. This perspective not only enriches understanding of the psychological adaptation mechanisms of frontline healthcare professionals under moral adversity but also provides a theoretical and practical basis for developing multi-level interventions aimed at safeguarding the well-being of emergency nurses, maintaining care quality, and supporting the sustainable development of the nursing profession.

## 5 Limitations

Although this study has made some contributions, there are still some limitations. First, the use of convenience sampling and a self-reported questionnaire may introduce response bias and limit the accuracy of the data. While strategies such as reverse-scored items and time-based filters were used to reduce this bias, its complete elimination is not guaranteed. Second, the study employed a cross-sectional design, which prevents causal inferences and does not allow examination of long-term outcomes. Third, the sample conducted across five tertiary hospitals in China, despite offering internal consistency in organizational structure, may limit generalizability due to the relatively homogeneous cultural and institutional context. Moreover, limited demographic and professional diversity among participants may further restrict the transferability of findings. Future research should consider more diverse samples, longitudinal designs, and cross-cultural comparisons to enhance the robustness and applicability of the results.

## 6 Practical implications

Despite these limitations, the findings of this study provide valuable insights for practice and future research. First, the results underscore the necessity of organizational-level interventions. Healthcare institutions, policymakers, and nurse managers should develop and implement policies that help improve working conditions—such as ensuring flexible and safe staffing, promoting work-life balance, and fostering a supportive team culture—to enhance nurses’ access to external protective resources (71). Second, the study highlights the importance of moral education and ethical training. Nurse managers should actively advance relevant training programs to strengthen nurses’ moral awareness, professional values, and ethical decision-making skills, thereby building stronger internal psychological resources (72). By integrating the JD-R model and the buffering hypothesis, we demonstrated how external resources (perceived social support) and internal resources (moral resilience) interact dynamically to buffer the impact of MI. This dual-resource chain model highlights the role of external support in fostering internal psychological resilience, suggesting that in practice, it is essential not only to provide adequate social support systems for emergency nurses but also to strengthen their internal capacity for moral resilience (73). In addition, future research should be conducted within a broader geographical and cultural context, utilizing longitudinal or mixed-method designs to validate and expand the theoretical model proposed in this study. Furthermore, targeted intervention strategies can be developed based on the findings of this research to alleviate the impact of MI on nurses’ work performance and mental health. This approach will provide valuable guidance for both theoretical advancement and practical application.

## 7 Conclusion

This study not only confirmed the significant negative impact of MI on emergency nurses’ HRPL but also demonstrated, for the first time, the critical mediating roles of perceived social support and moral resilience within this relationship. By empirically validating this dual-pathway mechanism, the study offers a novel theoretical perspective that integrates the JD-R model with the Buffering Hypothesis to explain how external and internal resources dynamically interact to counteract the detrimental effects of MI. These findings provide practical guidance for healthcare organizations and nurse managers to develop multi-level interventions, such as strengthening supportive workplace cultures, enhancing ethical education, and promoting individual resilience strategies to protect nurses’ mental well-being and sustain their productivity under high moral stress.

## Data Availability

The original contributions presented in the study are included in the article/Supplementary material, further inquiries can be directed to the corresponding author.

## Footnotes

^1^ www.wjx.cn
